# Actin- and clathrin-dependent mechanisms regulate interferon gamma release after stimulation of human immune cells with respiratory syncytial virus

**DOI:** 10.1186/s12985-016-0506-6

**Published:** 2016-03-22

**Authors:** Jop Jans, Hicham elMoussaoui, Ronald de Groot, Marien I. de Jonge, Gerben Ferwerda

**Affiliations:** Laboratory of Pediatric Infectious Diseases, Department of Pediatrics, Radboud Institute for Molecular Life Science, Radboud university medical center, P.O. Box 9101, 6500 HB Nijmegen, The Netherlands

**Keywords:** Respiratory syncytial virus, Cell entry, Innate immunity, Molecular biology, Monocytes, Interferon gamma, T cells

## Abstract

**Background:**

Respiratory syncytial virus (RSV) can cause recurrent and severe respiratory tract infections. Cytoskeletal proteins are often involved during viral infections, either for cell entry or the initiation of the immune response. The importance of actin and clathrin dynamics for cell entry and the initiation of the cellular immune response against RSV in human immune cells is not known yet. The aim of this study was to investigate the role of actin and clathrin on cell entry of RSV and the subsequent effect on T cell activation and interferon gamma release in human immune cells.

**Methods:**

Peripheral blood mononuclear cells and purified monocytes were isolated from healthy adults and stimulated in vitro with RSV. Actin and clathrin dynamics were inhibited with respectively cytochalasin D and chlorpromazine. T cell receptor signaling was inhibited with cyclosporin A. Flow cytometry was used to determine the role of actin and clathrin on cell entry and T cell activation by RSV. Enzyme-linked immunosorbent assays were used to investigate the contribution of actin and clathrin on the release of interferon gamma.

**Results:**

Cell entry, virus gene transcription and interferon gamma release are actin-dependent. Post-endocytic processes like the increased expression of major histocompatibility complex II on monocytes , T cell activation and the release of interferon gamma are clathrin-dependent. Finally, T cell receptor signaling affects T cell activation, whereas soluble interleukin 18 is dispensable.

**Conclusion:**

Analysis of cell entry and interferon gamma release after infection with RSV reveals the importance of actin- and clathrin-dependent signaling in human immune cells. Insights into the cellular biology of the human immune response against respiratory syncytial virus will provide a better understanding of disease pathogenesis and may prove useful in the development of preventive strategies.

## Background

Respiratory syncytial virus (RSV) is a negative-sense single stranded RNA virus of the family *Paramyxoviridae* and is a major burden on the current health care system. In healthy adults, RSV infections are limited to the upper respiratory tract, but remarkably do not generate long-term immunity [1]. In children and elderly, RSV can cause severe lower respiratory tract infections requiring admission to an intensive care unit in a small percentage of cases.

The first line of defense against RSV infection consists of epithelial cells. Upon infection, epithelial cells attract antigen-presenting cells, including dendritic cells and monocytes. Monocytes and macrophages are able to engulf pathogens leading to antigen-presentation. The monocytic cell is one of the major immune cell types that is susceptible to RSV infection and the role of monocytes and macrophages in the pathogenesis of RSV infections has been appreciated for decades [2–7]. During RSV infection in mice, the recruitment of monocytes from the bloodstream limits viral replication and reduces disease severity [8]. Viral particles can interact with receptors at the membrane of monocytes resulting in attachment, uptake and initiation of the immune response [9–11]. Under many circumstances, actin or clathrin are essential for receptor-mediated internalization [12–16]. Internalization can be regulated differentially dependent on the cell type. Uptake of transferrin occurs clathrin-dependent in macrophages and is not dependent on clathrin in epithelial cells [17]. Cell-specific differences in entry mechanisms between epithelial cells and fibroblasts have been shown for human cytomegalovirus [18]. Previous studies have studied the internalization of RSV in epithelial cells [19–22]. No data is available regarding cell entry of RSV in monocytes, which raises the question whether internalization of RSV occurs differentially in innate immune cells. After internalization, immune cells are involved in antigen-presentation, T cell activation and the production of cytokines like interferon gamma (IFN-γ). IFN-γ, a type II interferon, plays a critical role in the immune response against viral infections [23]. T cell activation may occur through cytokines like interleukin 18 (IL-18) or through stimulation of the T cell receptor (TCR). The relationship between cell entry, T cell activation and subsequent release of IFN-γ during RSV infection in primary human cells is unknown. Peripheral blood mononuclear cells (PBMCs) provide a useful model to investigate the impact of cellular pathways on antiviral immunity. PBMCs contain important cells that reflect the immune response against RSV like dendritic cells, monocytes and T cells [4, 24–27]. In this study, we aimed to investigate the regulation of IFN-γ by actin- and clathrin-dependent mechanisms after stimulation of human immune cells with RSV. For this, we used pharmacological inhibitors to inhibit actin and clathrin. Hereby, the contribution of actin- and clathrin-dependent processes on cell entry, T cell activation and induction of IFN-γ in primary human immune cells during RSV infection was studied.

## Results

### Cell entry and subsequent virus gene transcription of RSV in monocytes are actin-dependent

**Fig. 1:**
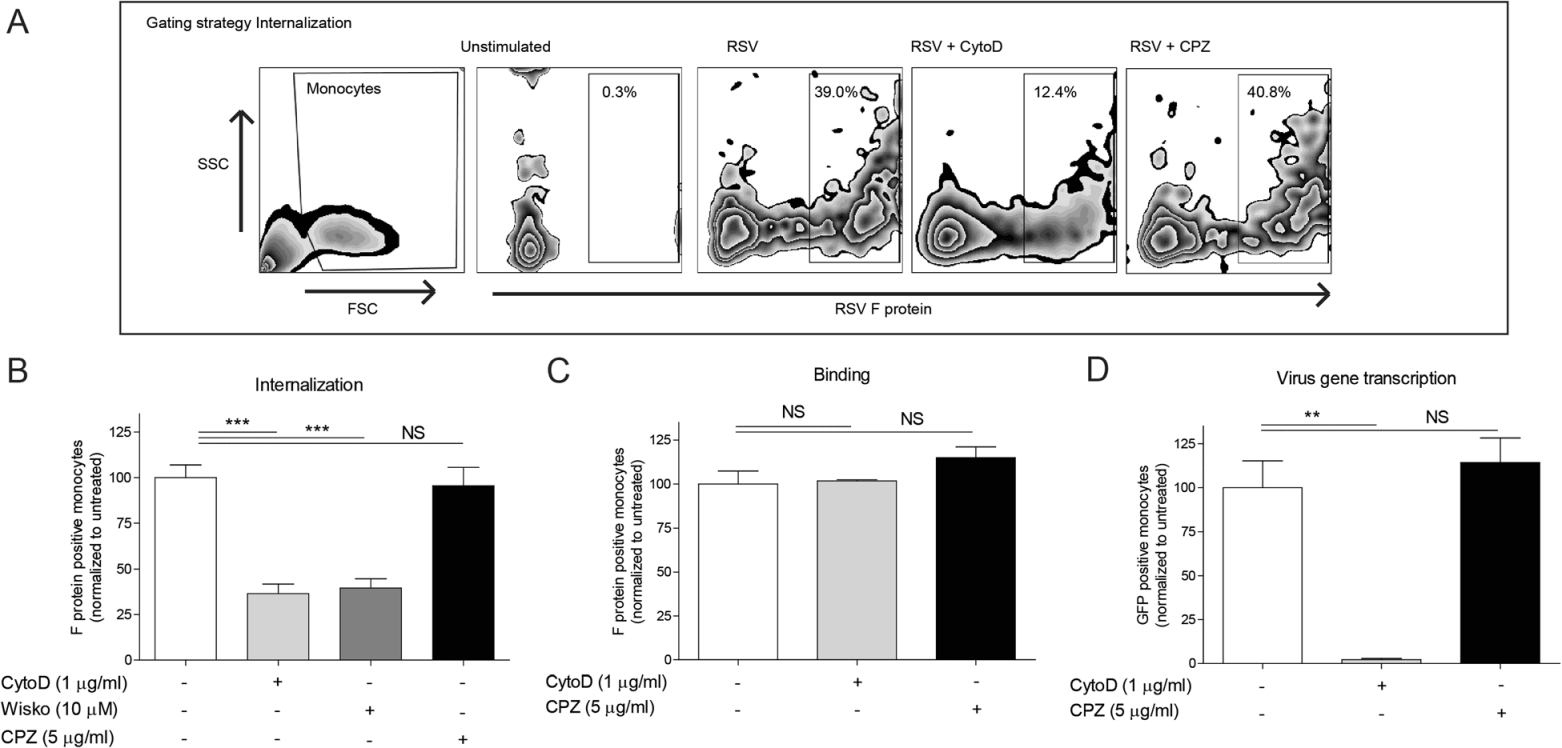
Cell entry and subsequent virus gene transcription of RSV in monocytes is actin-dependent. **a** Gating strategy to determine the amount of internalization of RSV in monocytes. **b** Monocytes were pre-treated with RPMI, CytoD (1 μg/ml), Wisko (10 μM) or CPZ (5 μg/ml). After pre-treatment, monocytes were incubated with RPMI or RSV at MOI 1 for 1 h at 37 °C and internalization was measured. **c** Monocytes were pre-treated with RPMI, CytoD (1 μg/ml), or CPZ (5 μg/ml). After pre-treatment, monocytes were incubated with RPMI or RSV at MOI 1 for 1 h at 4 °C and binding was measured. **d** PBMCs were pre-treated with RPMI, CytoD (1 μg/ml) or CPZ (5 μg/ml). After pre-treatment, PBMCs were incubated with RPMI or RSV at MOI 1 for 24 h and virus gene transcription was measured in monocytes. One-way analysis of variance with Bonferroni’s Comparison Test was used for statistical analysis. Data are normalized to untreated cells and depicted as mean ± SEM (*N* = 4-6). (**p < 0.01, ***p < 0.001) (NS = no significant difference between untreated and CytoD-treated cells or between untreated and CPZ-treated cells)

We first examined the dynamics of cell entry of RSV into CD14^+^ monocytes by using pharmacological inhibitors. Cytochalasin D (CytoD) and Wiskostatin (Wisko) have been used in previous literature to inhibit actin-dependent entry and chlorpromazine (CPZ) for clathrin-dependent entry [28–30]. A representative figure of the gating strategy to determine internalization of RSV into monocytes is shown (Fig. 1a). Disruption of actin filaments in monocytes with CytoD or Wisko significantly reduces the internalization of RSV whereas inhibition of clathrin with CPZ has no effect on the internalization indicating that cell entry is an actin-dependent process (Fig. 1b). Extracellular binding of RSV on membrane of monocytes occurred and is not reduced by pre-treatment of monocytes with CytoD or CPZ (Fig. 1c). To confirm the inhibitory effect of CytoD on cell entry, PBMCs were stimulated with RSV for 24 h and virus gene transcription was measured in monocytes. Treatment of PBMCs with CytoD abrogates virus gene transcription in monocytes whereas CPZ has no effect (Fig. 1d). Mean percentages of internalization, binding and virus gene transcription in untreated monocytes were respectively 29 %, 13 % and 4 %.

### IFN-γ release after RSV infection is actin- and clathrin-dependent

**Fig. 2:**
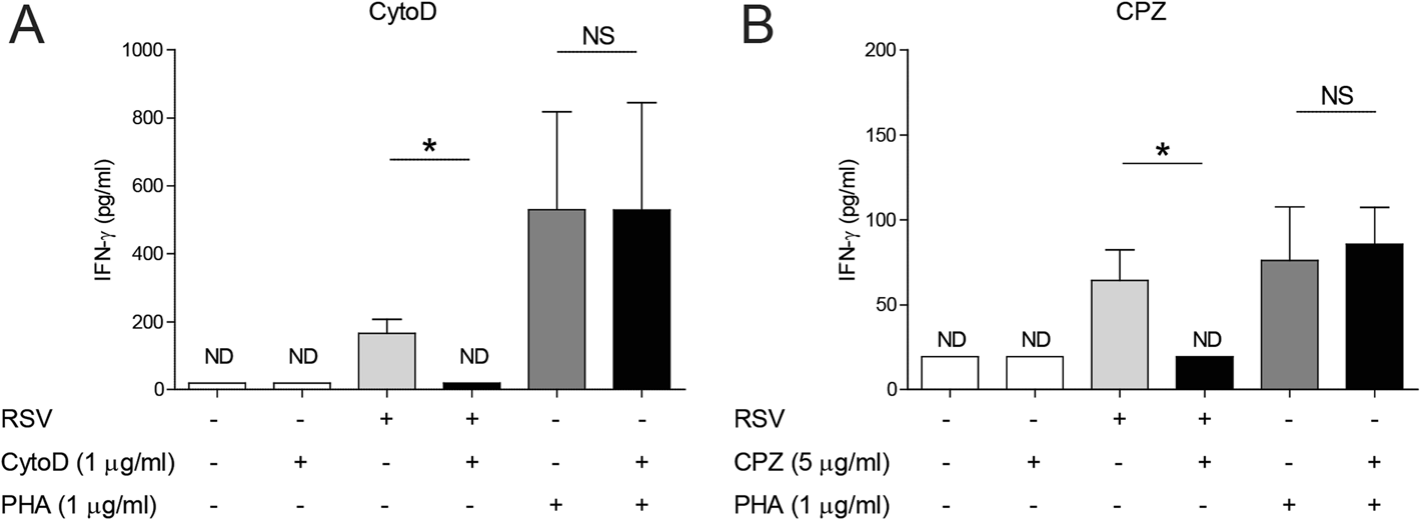
IFN-γ release after RSV infection is actin- and clathrin-dependent. **a**-**b** PBMCs were pre-treated with RPMI, CytoD (1 μg/ml) or CPZ (5 μg/ml). After pre-treatment, PBMCs were incubated with RPMI, RSV at MOI 1 or PHA (1 μg/ml) for 24 h and IFN-γ release was measured. Lower limit of detection: 20 pg/ml. Wilcoxon-signed rank test was used for statistical analysis. Data are mean ± SEM (*N* = 4-6). (*p < 0.05) (ND = non-detectable, NS = no significant difference between untreated and CytoD-treated cells or between untreated and CPZ-treated cells)

Previous literature shows that T cells are the main producers of IFN-γ in our model of PBMCs and stimulation with RSV [24]. To determine whether the adaptive immune response against RSV is actin-dependent, the IFN-γ release was measured after stimulation of PBMCs with RSV. Inhibition of actin with CytoD abrogates the RSV-induced IFN-γ release (Fig. 2a). Although CPZ has no effect on the internalization of RSV, inhibition of clathrin reduces the release of IFN-γ (Fig. 2b). Both CytoD and CPZ have no effect on T cell activation after stimulation with the non-specific T cell activator phytohaemagglutinin (PHA) (Fig. 2a–b). From this, we conclude that both cell entry and post-endocytic processes play a role in the adaptive immune response against RSV whereas mere binding of RSV to the cell membrane of monocytes is not sufficient to induce IFN-γ release.

### T cell activation and upregulation of MHC-II after RSV infection are clathrin-dependent

**Fig. 3:**
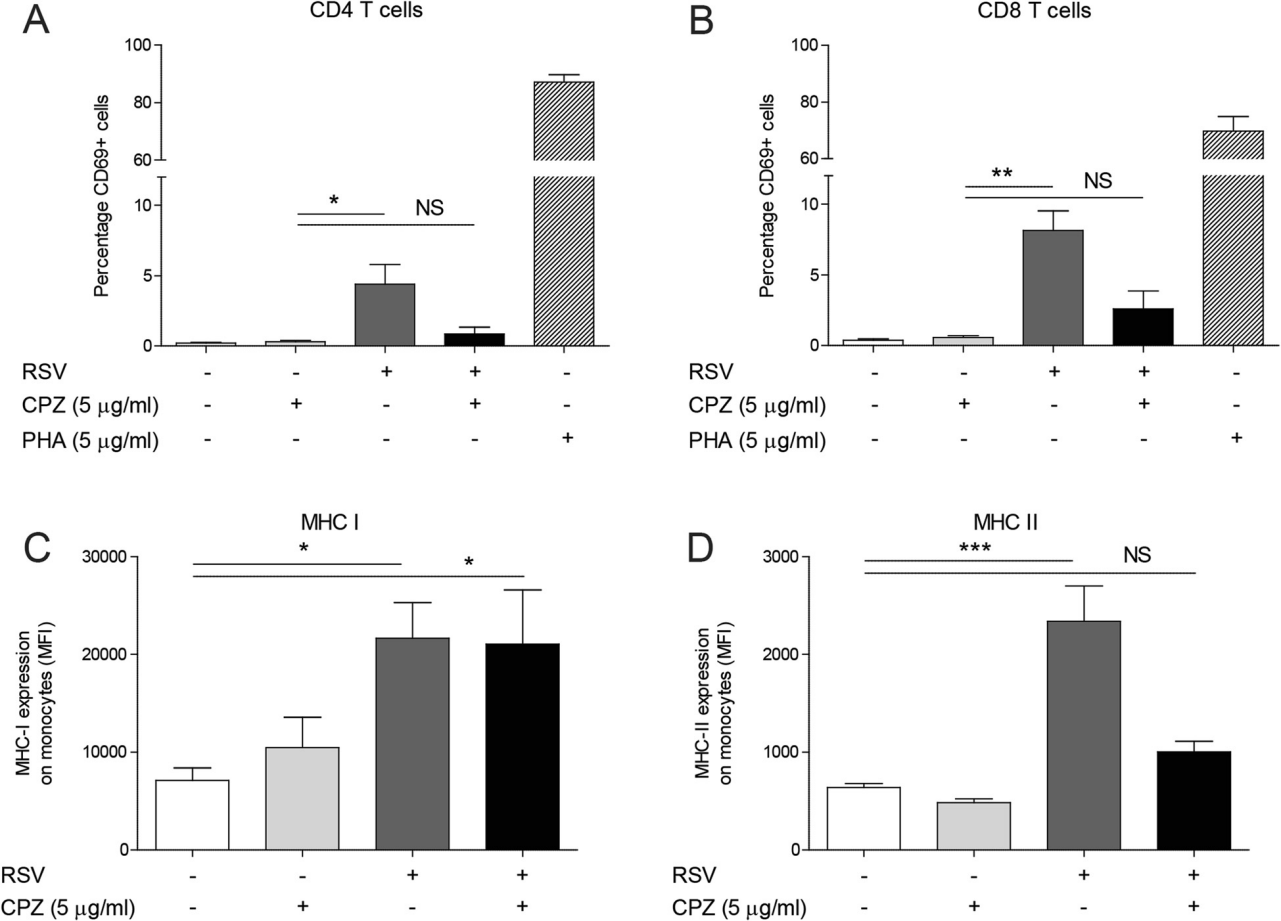
T cell activation and upregulation of MHC-II after RSV infection are clathrin-dependent. **a**-**d** PBMCs were pre-treated with RPMI or CPZ (5 μg/ml). After pre-treatment, PBMCs were incubated with RPMI or RSV at MOI 1 for 24 h. The effect of CPZ on (**a**) CD4 T cell activation, (**b**) CD8 T cell activation and (**c**) MHC-I and (**d**) MHC-II expression on monocytes cells was determined. Stimulation with PHA (5 μg/ml) was used as positive control for T cell activation (**a-b**). One-way analysis of variance with Bonferroni’s Comparison Test was used for statistical analysis. Data are mean ± SEM (*N* = 4). (*p < 0.05,**p < 0.01,***p < 0.001). (NS = no significant difference between unstimulated and RSV-stimulated cells)

To address the discrepancy between clathrin-independent internalization and clathrin-dependent IFN-γ release, we examined whether post-endocytic processes like T cell activation and upregulation of antigen-presenting molecules are clathrin-dependent. CD69 was used as an activation marker on T cells [31]. PHA was used as positive control for T cell activation. RSV induces activation of CD4 and CD8 T cells and pre-treatment of PBMCs with CPZ prevents T cell activation after RSV infection (Fig. 3a–b). To investigate whether the upregulation of antigen-presenting molecules is inhibited by CPZ, the expression levels of MHC-I and MHC-II on monocytes was determined. RSV induces a significant increase of MHC-I and MHC-II expression on monocytes (Fig. 3c–d). Upregulation of MHC-I expression is not affected by treatment of PBMCs with CPZ (Fig. 3c). The RSV-induced upregulation of MHC-II expression is not present after pre-treatment of PBMCs with CPZ indicating that upregulation of MHC-II is clathrin-dependent (Fig. 3d).

### IFN-γ release after RSV infection is dependent on T-cell receptor signaling

**Fig. 4:**
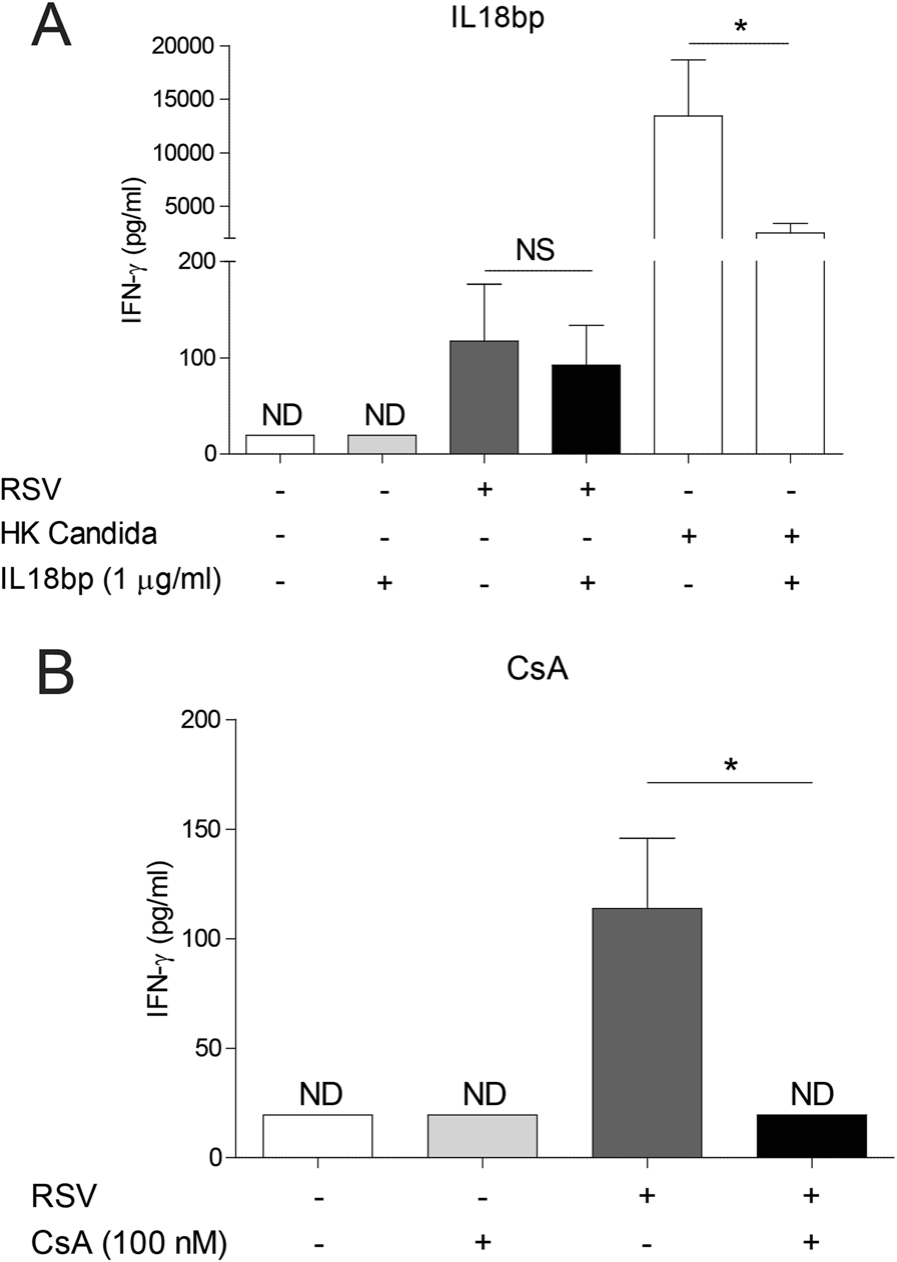
IFN-γ release after RSV infection is dependent on T-cell receptor signaling. **a** IL-18 bp was added to PBMCs simultaneously with RPMI, RSV at MOI 1 or heat killed Candida. IFN-γ release was measured after 24 h. **b** PBMCs were pre-treated with RPMI or CsA (100 nM). After pre-treatment, PBMCs were incubated with RPMI or RSV at MOI 1 for 24 h and IFN-γ release was measured. Lower limit of detection: 20 pg/ml. Wilcoxon-signed rank test was used for statistical analysis. Data are mean ± SEM (*N* = 4-5). (*p < 0.05) (ND = non-detectable) (NS = no significant difference between untreated and CPZ-treated cells)

To confirm the role of antigen-presenting molecules and thereby TCR signaling as its ligand, we evaluated whether IL-18, as a soluble T cell activator, or TCR signaling affects the IFN-γ release after RSV infection. Neutralization of IL-18 with IL-18 bp does not inhibit RSV-induced IFN-γ release. As a control, we show that IL-18 bp is able to significantly inhibit Candida-induced IFN-γ response, which is consistent with previous literature (Fig. 4a) [32]. The release of IFN-γ is inhibited when TCR signaling is blocked with cyclosporin A (CsA) (Fig. 4b).

## Discussion

Internalization and subsequent virus gene transcription of RSV in human monocytes and the induction of IFN-γ are actin-dependent. Although clathrin is not involved in the internalization of RSV, upregulation of MHC-II on monocytes, T cell activation and the release of IFN-γ after RSV infection are clathrin-dependent processes. Finally, T cell receptor activation contributes to the RSV-induced IFN-γ response whereas the production of IL-18 is dispensable.

Actin is required for cell entry of RSV in epithelial cells [19]. We have shown for the first time, that primary human monocytes also require actin to internalize RSV for subsequent virus gene transcription to occur. Actin plays an essential role in processes involving internalization, including receptor-mediated phagocytosis. For the Nipah virus, a paramyxovirus like RSV, infection induces the co-internalization of Nipah virus receptor ephrinB2 and, in general, many receptors are internalized in an actin-dependent manner [33–35]. Several receptors are involved in the recognition of RSV at the membrane, like Toll-like receptor 4 (TLR4), CD14 and nucleolin [9, 36]. Internalization of TLR4 and CD14 has been investigated upon bacterial ligand stimulation. In this process, internalization of TLR4 is dynamin-dependent and CD14 is actin-dependent [37, 38]. Clustering of nucleolin is dependent on actin [39]. Whether actin-dependent clustering of nucleolin occurs after RSV infection and whether it is necessary for internalization is unknown. Our finding that intact actin filaments are required for internalization of RSV in monocytes narrows the scope of potential receptors that co-occur with cell entry and are required for internalization. Besides cell entry, intact actin is required for virus filament formation, viral transmission and the production of cell-associated infectious virus by epithelial cells [40–43]. We observed an inhibitory effect of CytoD on the release of IFN-γ during an incubation period of 24 h. Inhibition of post-endocytic processes like virus filament formation and transmission could therefore explain the observed reduction of IFN-γ after treatment with CytoD. However, in our model of PBMCs, monocytes exhibit abortive infection as no replicating virus is detected in the supernatant of PBMCs stimulated with RSV (data not shown). This is in line with the abortive infection of RSV in alveolar macrophages [44]. Therefore, the role of actin on virus filament formation and viral transmission may be applicable to epithelial cells but most likely would not play a role in our model.

After inhibition of actin with CytoD or Wisko, approximately 30 % of the monocytes still remain positive for RSV F protein. Permeabilization of the cells in our assay will not exclude extracellular staining of RSV F protein and therefore the remaining RSV-positive monocytes after treatment could reflect extracellular binding of RSV. Because CytoD does not influence the extracellular binding of RSV, we conclude that actin dynamics are involved in the internalization of RSV by monocytes. Actin-independent processes could also explain the incomplete prevention of internalization by CytoD. Fusion of the F protein of RSV with the cell membrane could result in entry of viral proteins and might act independently of actin filaments. Clathrin has previously been implicated for internalization of RSV in an epithelial cell line after an incubation period of 20 h [20]. Contrary, others have shown that internalization of RSV after 1 h is not clathrin-dependent [19]. The authors conclude that discrepancies between these studies may arise due to a difference in experimental design. The incubation period of 1 h in our study excludes inhibition of post-endocytic processes like viral replication and cell-to-cell transmission. In addition, differences between epithelial cells and monocytic cells have been described previously for internalization of nanoparticles and may explain our results compared to epithelial cells [17]. The role of cellular mechanisms on T cell activation can be studied in our model of PBMCs. Besides internalization, inhibition of actin dynamics reduces the IFN-γ response. These data suggest that, at least in part, the intracellular compartment is involved in the induction of an adaptive response and binding of RSV to the outer membrane is not sufficient to induce IFN-γ release. These results are in line with previous data indicating that intact RSV particles are not able to signal via membrane-associated receptors such as TLR4 [45].

In our study, inhibition of clathrin has no effect on the internalization of RSV by monocytes, but effectively reduces the induction of IFN-γ. CPZ at 5 μg/ml was the highest tolerable concentration without inducing cytoxicity (data not shown). Our data suggest that activation of T cells by RSV is clathrin-dependent. Calabia-Linares et al. observed the importance of clathrin in the formation of the immunological synapse between T cell and antigen-presenting cells [46]. Possibly, inhibition of clathrin reduces the IFN-γ release due to improper formation of the immunological synapse and thereby inefficient T cell activation. Less than 1 % of the T cells in the peripheral blood are RSV-specific T cells [47, 48]. Therefore, the relative high percentage of 5-10 % CD69^+^ T cells after stimulation with RSV in our experiments most likely indicates that a general activation of T cells occurs. We demonstrate that clathrin plays an essential role in the upregulation of RSV-induced MHC-II expression on monocytes . The requirement of clathrin for MHC-II trafficking has been observed previously [49]. The role of MHC-II in the induction of IFN-γ is strengthened by the observation that inhibition of TCR signaling, as a ligand for MHC-II, reduces the IFN-γ response, whereas inhibition of IL-18 has no effect. The combination of reduced T cell activation and MHC-II expression suggests that clathrin plays a role in the interplay between innate and adaptive immunity against RSV.

The simplification of in vivo RSV infections using our model of PBMCs could be a limitation of the study. Although others have shown that monocytes can be become infected with RSV and serve as an in vitro model, alveolar macrophages or inflammatory macrophages are the most likely target of infection in vivo [4]. Therefore, there could be differences in the immune response in vivo compared to our model. Pre-treatment of isolated monocytes with the inhibitors and performing a subsequent co-culture with autologous T cells could be considered as an alternative instead of simultaneous culture of all cells present in PBMCs. However, during RSV infection in vivo, multiple immune cells, including monocytes, natural killer cells and T cells are recruited to the lung tissue [8]. Our model can give more insights in the interplay between RSV and different immune cells that are present during RSV infection.

In conclusion, this study underlines the importance of actin and clathrin dynamics during RSV infections for cell entry and T cell activation. Currently, there is no effective treatment or vaccine against RSV infections. Understanding the different aspects of RSV disease pathogenesis and immunity, from early virus-cell interactions to final induction of the adaptive immune response may contribute to the development of novel therapies and effective vaccines.

## Conclusions

The aim of this study was to investigate the role of actin and clathrin on cell entry of RSV and the subsequent effect on T cell activation and release of IFN-γ in human immune cells. Cell entry in human monocytes, virus gene transcription and IFN-γ release are actin-dependent. Post-endocytic processes like the increased expression of MHC-II on monocytes , T cell activation and the release of IFN-γ are clathrin-dependent. Finally, T cell receptor signaling affects the release of IFN-γ, whereas soluble interleukin 18 is dispensable. Insights into the cellular biology of the human immune response against RSV will provide a better understanding of disease pathogenesis.

## Methods

### Pharmacological inhibitors

Actin-dependent pathways were inhibited by pre-treatment of cells with CytoD (1 μg/ml) or Wisko (10 μM) and clathrin-dependent pathways were inhibited by CPZ (5 μg/ml) (Sigma-Aldrich). CsA (Sigma-Aldrich) and IL-18 bp (R&D Systems, United Kingdom) were used to respectively inhibit T cell receptor signaling and IL-18. No cytotoxic effect determined as Annexin V+/7-AAD+ cells was observed when using the indicated concentrations of the inhibitor (data not shown).

### Cell isolation

After obtaining informed consent, peripheral blood mononuclear cells (PBMCs) from healthy adults were isolated using Lymphoprep and isolation of monocytes from the PBMC fraction was performed using an indirect negative selection magnetic labeling kit (Monocyte Isolation Kit II human; Miltenyi Biotec). Each 96-well plate was filled with 5 x 10^5^ PBMC per well or 1x10^5^ isolated monocytes per well. The study was approved by the committee on Research involved Human Subjects of the Radboudumc.

### Culture of RSV

RSV A2 containing an additional transcription unit encoding GFP (rgRSV) was cultured as previously described [50]. rgRSV was cultured on HeLa cells (ATCC, CCL-2) in Dulbecco’s minimum essential medium (DMEM) with 10 % fetal calf serum (FCS) and 1 % penicillin/streptomycin. Near-confluent HeLa cells were infected with rgRSV and incubated for three days at 37 °C. Cells were scraped and the suspension was centrifuged to remove cellular debris. rgRSV was ultracentrifuged on a sucrose cushion for purification and titrated on HeLa cells. Confluent HeLa cells (80-90 %) were infected with fivefold viral dilutions for 20-22 h. Virus titer was determined by counting wells with ≥10 and ≤100 infected cells/view (CKX41 microscope; Olympus, Tokyo, Japan). rgRSV was snapfrozen and stored at −80 °C until use.

### Internalization and binding assay

PBMCs or isolated monocytes were treated with CytoD (1 μg/ml), Wisko (10 μM) or CPZ (5 μg/ml) for 30 min at 37 °C. For quantification of cell entry, monocytes were permeabilized with CytoFix/Perm (BD Biosciences) after incubation with RSV at MOI 1 (5 x 10^5^ IU/well) for 1 h at 37 °C. Cells were incubated for 30 min on ice with mouse anti-respiratory syncytial virus fusion protein antibody (Ab24011; Abcam). Goat anti-mouse IgG PE (BD Pharmingen) was used as secondary antibody. For the quantification of RSV binding, co-incubations of PBMCs with RSV were performed for 1 h at 4 °C and cells were fixed with 1 % paraformaldehyde before staining.

### T cell activation and MHC expression

PBMCs were treated with CPZ (5 μg/ml) for 30 min at 37 °C. PBMCs were stimulated with RSV at MOI 1 or with PHA (5 μg/ml) for 24 h. CD69 expression, as T cell activation marker, was measured with flow cytometry. For gating, CD3 and CD4 or CD8 positive T cells were selected and the percentage of CD69 positive cells was determined. After pre-treatment with CPZ (5 μg/ml), PBMCs were incubated with RSV at MOI 1 for 24 h and the expression of MHC-I and MHC-II on monocytes was determined. For gating, CD14 positive cells were selected and the geometric mean fluorescence intensity (MFI) of MHC-I and MHC-II was determined.

### Flow cytometry

Cell surface markers were stained with CD14 V500, CD14 AF647, CD3 V500, CD8 APC-H7, CD8 PE, CD4 PerCP-Cy5.5, CD69 Pe-Cy7, HLA-ABC PE and HLA-DR PE (BD Pharmingen). For quantification of RSV, mouse anti-RSV F protein (Ab24011; Abcam) and goat anti-mouse IgG PE (BD Pharmingen) were used. Virus gene transcription in monocytes was determined by gating CD14 positive cells as monocytes and calculating the percentage of GFP positive cells. Cytoxocity was determined by calculating Annexin V+/7-AAD+ cells (PE Annexin V Apoptosis Detection Kit I, BD Pharmingen). Events were acquired on an LSR II flow cytometer and analyzed using FlowJo.

### In vitro cytokine production

PBMCs were treated with CytoD (1 μg/ml), CPZ (5 μg/ml) or CsA (100 nM) for 30 min at 37 °C. After pre-treatment, cells were stimulated with RSV at MOI 1, PHA (1 μg/ml) or heat-killed Candida albicans (1 x 10^6^/well) for 24 h. IL-18 bp (1 μg/ml) was added simultaneously with RSV. Concentration of interferon gamma were measured in the cell supernatants by enzyme-linked immunosorbent assays (ELISA) (Sanquin Blood Supply, the Netherlands) with a lower limit of detection of 20 pg/ml.

## Abbreviations

CPZ: chlorpromazin
CsA: cyclosporin A
CytoD: cytochalasin D
ELISA: enzyme-linked immunosorbent assay
IFN-γ: interferon gamma
IL-18: interleukin 18
IL-18 bp: interleukin 18 binding protein
MFI: geometric mean fluorescence intensity
MHC: major histocompatibility complex
MOI: multiplicity of infection
PBMC: peripheral blood mononuclear cells
PHA: phytohaemagglutinin
RSV: respiratory syncytial virus
TCR: T cell receptor
TLR4: toll-like receptor 4
Wisko: Wiskostatin
